# Neural progenitor cells from an adult patient with fragile X syndrome

**DOI:** 10.1186/1471-2350-6-2

**Published:** 2005-01-14

**Authors:** Philip H Schwartz, Flora Tassone, Claudia M Greco, Hubert E Nethercott, Boback Ziaeian, Randi J Hagerman, Paul J Hagerman

**Affiliations:** 1 National Human Neural Stem Cell Resource, Children's Hospital of Orange County Research Institute, Orange, CA, USA; 2 Stem Cell Research, Children's Hospital of Orange County Research Institute, Orange, CA, USA; 3 Developmental Biology Center, School of Biological Sciences, University of California, Irvine, Irvine, CA, USA; 4 Departments of Biochemistry and Molecular Medicine, University of California, Davis, School of Medicine, Davis, CA, USA; 5 Departments of Pathology, University of California, Davis, School of Medicine, Davis, CA, USA; 6 Departments of Pediatrics, University of California, Davis, School of Medicine, Davis, CA, USA; 7 M.I.N.D. Institute, University of California, Davis, School of Medicine, Davis, CA, USA

## Abstract

**Background:**

Currently, there is no adequate animal model to study the detailed molecular biochemistry of fragile X syndrome, the leading heritable form of mental impairment. In this study, we sought to establish the use of immature neural cells derived from adult tissues as a novel model of fragile X syndrome that could be used to more fully understand the pathology of this neurogenetic disease.

**Methods:**

By modifying published methods for the harvest of neural progenitor cells from the post-mortem human brain, neural cells were successfully harvested and grown from post-mortem brain tissue of a 25-year-old adult male with fragile X syndrome, and from brain tissue of a patient with no neurological disease.

**Results:**

The cultured fragile X cells displayed many of the characteristics of neural progenitor cells, including nestin and CD133 expression, as well as the biochemical hallmarks of fragile X syndrome, including CGG repeat expansion and a lack of FMRP expression.

**Conclusion:**

The successful production of neural cells from an individual with fragile X syndrome opens a new avenue for the scientific study of the molecular basis of this disorder, as well as an approach for studying the efficacy of new therapeutic agents.

## Background

Fragile X syndrome, the leading heritable form of mental impairment [1,2], is generally caused by the expansion of a trinucleotide (CGG) repeat element in the fragile X mental retardation 1 (*FMR1*) gene to greater than 200 repeats (full mutation) [3]. Such expansions generally lead to methylation-coupled gene silencing [4,5] and the consequent absence of the *FMR1 *protein (FMRP), an RNA-binding protein that is important for neural development and plasticity [6,7].

Although great strides have been made from animal models in our understanding of the neuropathology of fragile X syndrome [8-13], there is, at present, no adequate animal or cell model to study the detailed molecular biochemistry of the *FMR1 *gene. The absence of a suitable model system is a consequence of the inability to clone full mutation alleles, and because no animal system has been found that carries native, full mutation *FMR1 *alleles. Thus, all animal or cell model systems for fragile X syndrome are based on *FMR1 *(homolog) knock-out constructs. While these models qualitatively recapitulate some of the features of the fragile X syndrome phenotype, they do not address any of the potential consequences of the expanded methylated CGG repeat.

As with most disorders of the human nervous system, it has been impossible to directly study the detailed cellular pathogenic mechanisms that underlie fragile X syndrome, due to the absence of a suitable (human) cell model. However, this barrier may be overcome through the use of neural progenitor cells, which comprise relatively undifferentiated populations of cells in the central nervous system (CNS) that give rise to the broad array of specialized cells, including neurons and glial cells. Long thought to be an exclusive component of the developing CNS, these cells have been shown to exist in the adult CNS [14-19]. Recent research, demonstrating that these cells can be isolated and cultured [14,18,20-22] has raised the prospect of using neural progenitor cells as a human cell-appropriate (neuronal and astrocytic) model system for the detailed study of the molecular biology of fragile X syndrome.

Importantly, we have previously shown that neural progenitor cells can be harvested from adult post-mortem brain tissue [18,20], which represents a singular advantage over the use of neural stem cells from fetal sources. In particular, since there is a broad spectrum of clinical involvement in fragile X syndrome [2], the study of neural stem cells from individuals of known phenotype provides us with a closer coupling of the genotype with the clinical phenotype.

In the current report, we describe the successful culturing of neural progenitor cells from an adult male with fragile X syndrome, and some of the characteristics of these cells. We further demonstrate initial efforts to differentiate these progenitor cells into both neuronal and astrocytic lineages. As expected, expression of FMRP is substantially reduced relative to its expression in neural cell culture from an unaffected control.

## Methods

### Autopsy, brain harvest, and tissue cryopreservation

Prior to tissue acquisition by the authors, informed consent for the donation of tissues was obtained under the auspices of the protocol for the National Human Neural Stem Cell Resource. This protocol is approved by Institutional Review Board (IRB) of Children's Hospital of Orange County, and follows a protocol approved by the UC Davis School of Medicine IRB. All tissues were acquired in compliance with NIH and institutional guidelines. Two patients were used for the present study: a patient with fragile X syndrome and a patient with no neurogenetic disease.

The autopsy followed standard procedures as described [20]. The periventricular zone in the area of the head of the caudate nucleus regions was identified and dissected from the appropriate brain sections. Brain region specimens were then placed in separate Petri dishes and rinsed three times with DGA (see below). Tissues were minced with sterile scalpel blades, triturated in DGF (see below) containing 10% DMSO, taken to -80°C overnight in controlled-rate freezing containers, and then transferred to liquid nitrogen Dewars for long term storage.

### Pathology

Portions of fresh brain, heart, and testicular tissue were received, and fixed in 10% formalin for 10 days prior to sampling and processing for paraffin sections in standard fashion. All tissue sections were stained with hematoxylin and eosin, with cardiac valves and aorta additionally stained for elastin and mucin. All sections were examined by standard light microscopy.

### Cell culture

The base medium was a high glucose 1:1 DMEM:F12 (Irvine Scientific). The basal medium (DGA) used for all other media was the base medium containing glutamine, penicillin, streptomycin, gentamicin, ciprofloxacin, and amphotericin as previously described [20]. Medium (DGF) used for all washes consisted of DGA containing 20% fetal bovine serum.

All procedures were performed as previously described [20], with modifications. Tissues were quickly thawed, diluted by drop-wise addition and agitated in 10 volumes of DGF, then further dissociated by trituration and three washes in DGF with centrifugation – no enzymatic digestion was used. Whole tissue homogenates were plated directly on fibronectin-coated tissue culture plates (6-well, tissue culture treated, Falcon) in primary growth medium (PGM) composed of DGF containing 10% BIT 9500 (Stem Cell Technologies), 40 ng/mL basic fibroblast growth factor (FGF-2; InVitrogen), 20 ng/mL epidermal growth factor (EGF; InVitrogen), and 20 ng/mL platelet-derived growth factor-AB (PDGF-AB; Peprotech). Plates had been previously incubated with 200 uL/cm^2 ^of fibronectin (5 ug/mL; Sigma) overnight at 37°C, the fibronectin solution aspirated, and the plates allowed to air dry before the introduction of tissue homogenates.

Approximately 300 mg fresh tissue was subjected to mincing and trituration, and the resulting crude tissue homogenate plated into six wells of a fibronectin-coated six-well tissue culture plastic plate (≈60 cm^2 ^total surface area).

After plating, 50% of the medium was replaced, 3 times weekly. Non-adherent cells and debris from the removed supernatant were pelleted by centrifugation and re-introduced into the cultures together with the fresh medium. After 7 days in culture, plates were agitated by sharp rapping with a marking pen and 100% of the culture medium and non-adherent material was removed. Fifty percent of the volume removed was replaced with fresh medium, while the removed medium was centrifuged to pellet cell debris and non-adherent cells and to recover conditioned medium as supernatant. Fifty percent, by volume, of the conditioned medium was then returned to the original plates. The pellet, containing the non-adherent fraction, was resuspended in 50% conditioned medium and 50% fresh medium, by volume, and then transferred to a fresh fibronectin-coated 6-well plate. After one week, the procedure was repeated, except that the non-adherent fraction was discarded. In this way, an additional population of cells was recovered from the non-adherent fraction. All the cells were eventually combined to form a single population of cultured cells.

At near confluence, cultures were passaged by lifting with a solution of Cell Dissociation Buffer (GIBCO) supplemented with trypsin. The cells were washed twice with DGF and plated in 1:1 (conditioned:fresh) medium into a fibronectin-coated T75 flask. Thereafter, and at approximately one week intervals, cells were lifted and similarly plated into twice the surface area from which they were removed. After the cells had reached a confluent surface area of 600 cm^2^, the medium from one T75 flask was exchanged with GM (PGM without serum), and these cells were cultured for two weeks before immunocytochemical analysis, or differentiation and immunocytochemical analysis, as previously described [20].

### Immunocytochemistry

Immunocytochemistry was performed as previously described [20]. Primary antibodies and dilutions were used as follows: nestin (1:100; mouse; Chemicon), type III β-tubulin (Tuj20; 1:100; mouse; Chemicon), MAP2ab (1:250; mouse; Sigma), GFAP (1:500, guinea pig; Advance Immuno), CD133-APC (1:100; mouse; Miltenyi), NCAM (1:100, rabbit, Chemicon), fusin (1:100, mouse, Chemicon), and FMRP (1:100; mouse; Chemicon). Coverslips were mounted with Prolong^® ^Antifade Kit (Molecular Probes, Eugene, OR). Some cells were stained with 4',6-diamidino-2-phenylindole (DAPI, Sigma) before being rinsed and mounted. Pictures were imaged on an Olympus IX70 Microscope and digitally photographed via a Microfire digital camera (Optronics, Goleta, CA) using Image Pro Plus 4.5 with AFA plugin 4.5 software.

### Molecular studies

#### DNA analysis

Genomic DNA was isolated from approximately 5 × 10^6 ^neural progenitor cells and from post-mortem sections of about 500 mg of brain tissue using standard methods (Puregene Kit; Gentra). For Southern blot analysis, 10 μg of isolated DNA were digested with *Eco*RI and *Nru*I. The *FMR1 *genomic probe StB12.3, labeled with Dig-11-dUTP by PCR (PCR dig synthesis Kit, Roche Diagnostics), was used in the hybridization, as described in Tassone et al. [31]. Genomic DNA was also amplified by PCR using primers c and f [32]; PCR products were detected using a digoxygenin-end-labeled oligonucleotide probe (CGG)_10_. Southern blot and PCR analyses were both carried out using an Alpha Innotech FluorChem 8800 Image Detection System.

#### FMR1 mRNA expression levels

Total RNA was isolated from approximately 1 × 10^6 ^neural progenitor cells and from post-mortem brain tissue using standard methods (Purescript, Gentra Inc. and Trizol). Reverse transcriptase reactions and quantitative fluorescence RT-PCR, using specific primers and probe set for the *FMR1 *gene and the control gene (β-glucoronidase; *GUS*), were carried out as described in Tassone et al. [33].

## Results

### Clinical history

JS was a 25-year-old man with fragile X syndrome. His history included motor and language delays in childhood, with walking at 20 months, phrases at three years, and sentences at 6 years of age. He was diagnosed with fragile X syndrome at 11 years of age, by cytogenetic testing. His behavior included hyperactivity, anxiety, shyness, poor eye contact, hand flapping, finger biting, and perseverative speech. At age 24, cognitive testing with the WAIS III demonstrated a verbal IQ of 58, performance IQ of 51, and full scale IQ of 51. He did not have autism; childhood autism rating scale (CARS) score was 28.5 (below autism range).

Previous medical history included severe mitral valve prolapse; echocardiography revealed moderate thickening and redundancy of both mitral leaflets, with central mitral regurgitation (grade 2 to 3+ by Doppler), mild tricuspid regurgitation, and moderate left ventricular and mild left atrial enlargement. Upon physical examination at age 24, JS had a long face, prominent ear pinna, high arched palate, and macroorchidism with testicular volume of 60 ml bilaterally. Blood pressure was 142/74, and a grade III/IV systolic and diastolic murmur with click was heard on examination. He died unexpectedly at age 25, presumably due to a cardiac arrhythmia secondary to mitral valve prolapse.

### Pathology

A complete autopsy was performed 16 hours after death. Abnormal findings included increased brain weight (1600 gm; normal, 1440 ± 20 g), an enlarged heart, (400 gm; normal, 349 ± 40 g), with features of mitral valve prolapse (myxomatous thickening of both leaflets; "hooding" of the posterior leaflet), and increased testicular weight of 73 g (normal average, 25 g). Our laboratory did not receive the intact brain, but portions of it, therefore detailed gross and histological evaluation was not possible. In particular, neither the hippocampus nor the amygdala was available for histopathological examination. Evaluation of available brain sections showed diffuse, acute early ischemic damage corresponding to the manner of death; no intraneuronal inclusions were seen, nor was there spongiosis of white matter in the cerebrum, cerebellum, or middle cerebellar peduncles. Cerebellar folia showed moderate patchy absence of Purkinje cells; because of the manner of death, loss due to ischemic damage cannot be ruled out. However, the majority of Purkinje cells present appeared histologically normal.

### Cell culture

**Figure 1:**
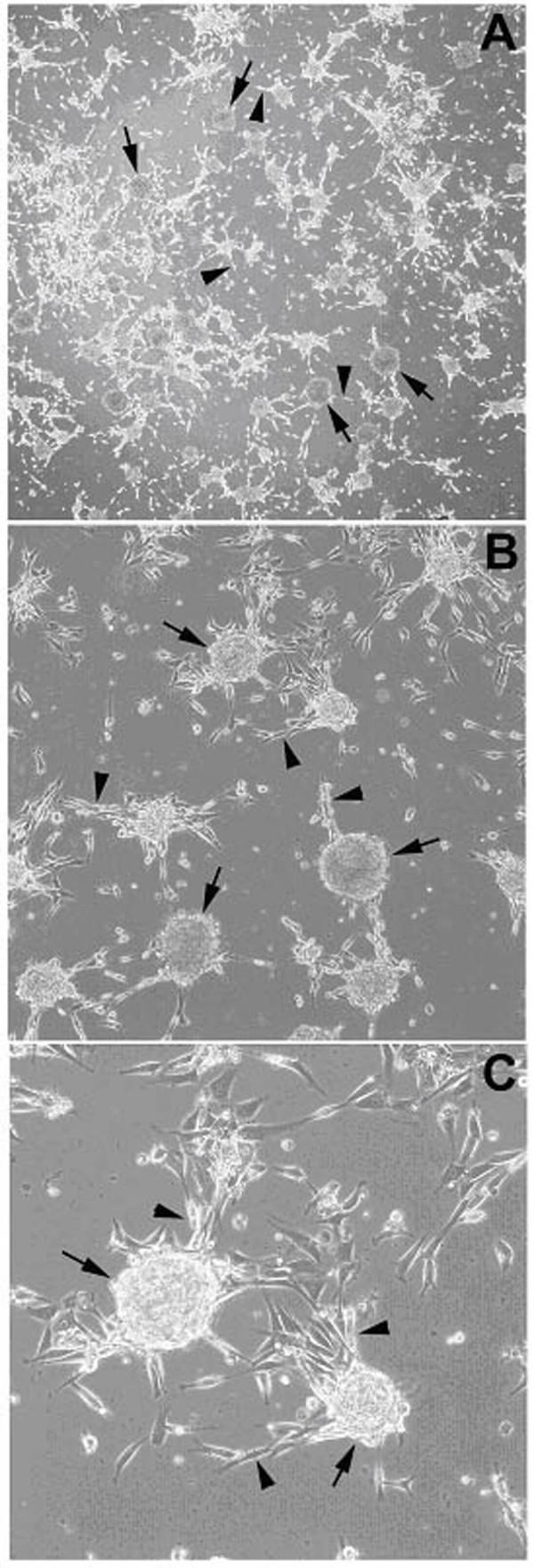
**Phase-contrast photomicrographs of fragile X progenitor cells **The figure shows clusters/spheres during the initial stages (2–3 days after plating) of adherence to a fibronectin substrate. (A) 4×; (B) 10×; (C) 20×. Confluent serum- and growth factor-expanded cultures were serum deprived for one week in the presence of growth factors, then lifted with enzyme-free buffers and transferred to new plates with no fibronectin substrate. After growing the resulting clusters/spheres for two weeks, the clusters/spheres were transferred to new fibronectin-coated plates. Clusters/spheres (black arrows) are abundant and are seen adhering to the substrate. Cells (black arrowheads) can be seen streaming from the spheres and spreading out on the substrate.

Minced brain tissue, derived from the periventricular zone in the area of the head of the caudate nucleus and maintained under proliferation conditions in serum-containing medium with growth factors, yielded viable cells that formed an adherent monolayer on fibronectin-coated plates. When lifted and plated without serum or fibronectin substrate, the cells grew in suspended clusters/spheres (Figure 1), similar to previously described neurospheres [20]. The morphology within the adherent population was variable and included small, rounded profiles, medium-sized bipolar and spindle-shaped profiles, and larger cells with polygonal and multipolar morphologies. Once a robust primary culture had been established in the six-well plates (approximately one month after plating of tissue homogenates), the cultures were passaged into T75 flasks approximately once per week until 600 cm^2 ^of confluent adherent cells had been produced. Cells were then further expanded under serum-free conditions for immunocytochemical analysis, or under growth-factor-free conditions for biochemical analysis.

Although the methodology used was similar to that previously reported [20], four conditions employed in the current work are noteworthy. (*i*) The cells were grown from cryopreserved, rather than fresh, tissue. This modification allows pathologists with no local access to a stem cell culture laboratory to preserve tissues for later stem cell harvest at a remote collaborating laboratory. (*ii*) No enzymatic digestion, only trituration, was used to generate the crude tissue homogenates. Preliminary studies showed that enzymatic digestion, typically used with fresh tissue, adversely affected our ability to harvest living cells from cryopreserved tissues. (*iii*) Serum (20%) was maintained in the culture until it was expanded to 600 cm^2 ^of confluent adherent cells, after which the cells were cultured in serum-free medium. Preliminary studies indicated that, unlike cells harvested from fresh tissues, cells harvested from cryopreserved tissues required application of serum for a longer time in culture to sustain a sufficient rate of proliferation. (*iv*) The non-adherent fraction was transferred to new fibronectin-coated plates after one week. Preliminary studies showed that the non-adherent fraction from cryopreserved tissues retained a significant population of viable cells for a longer period of time than that from fresh tissue. These plates were cultured for an additional week before the remaining non-adherent fraction was finally discarded. Cells grown from both sets of plates were combined for expansion.

### Immunocytochemistry

**Figure 2:**
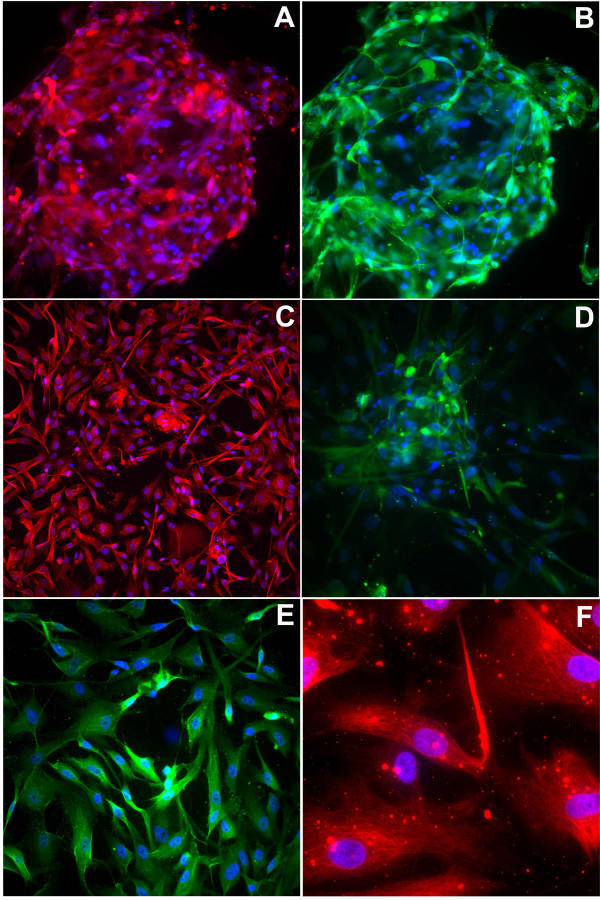
**Staining of fragile X progenitor cells, grown under expansion conditions **In all panels, nuclei are stained with DAPI (blue), while the second color represents antibody staining as follows: (A) multipotential neural progenitor lineage marker, CD133 (red); (B) neural cell adhesion molecule, NCAM (green); (C) CXCL12 (SDF-1) cytokine receptor, CXCR4 (fusin, CD184, red); (D) β-III-tubulin (green); (E) the glial fibrillary acidic protein, GFAP (green); (F) nestin. (100×).

**Figure 3:**
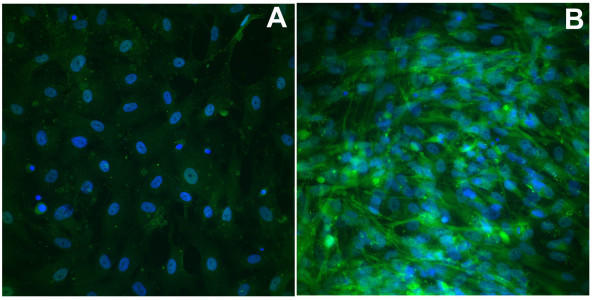
**Markedly reduced staining with anti-FMRP antibody of neural progenitor cells from a fragile X patient **FMRP staining (green) is greatly reduced in the fragile X derived cells (A) relative to progenitor cells from an unaffected control (B). Cell nuclei counterstained with DAPI (blue); both panels, 40×.

Immunocytochemical analysis of fragile X neural progenitor cells grown under expansion conditions demonstrated the expression of a range of developmental and mature neural markers (Figure 2). Many of the current results are similar to previous findings with control human neural progenitor cell cultures [20]. In particular, the distribution of the multipotential neural progenitor lineage marker, CD133, the neuroepithelial marker nestin, and the neural cell adhesion molecule, NCAM, are found to be widespread in these cultures, consistent with earlier observations of immunocytochemistry and flow cytometry in control human neural progenitor cells [20,23,24]. The CXCL12 (SDF-1) cytokine receptor, CXCR4 (fusin, CD184), and the glial neurofilament marker, glial fibrillary acidic protein (GFAP), are also widely expressed in the fragile X neural progenitor cell cultures, in agreement with a previous report [20]. The expression of β-III-tubulin, a mature neuronal marker, is restricted to subpopulations of cells, again consistent with control human neural progenitor cells [20]. Finally, the primitive neuroepithelial (intermediate filament) markers, nestin and CD133, seen in the proliferating neural progenitor cells (Figure 2), disappear under differentiation conditions (data not shown). Importantly, FMRP staining is markedly reduced in the fragile X neural progenitor cells (Figure 3) compared to control cultures.

### Molecular studies

Southern Blot analysis of DNA isolated from brain tissue (frontal cortex) showed the presence of a mosaic pattern. Specifically, full mutation alleles were present in 82% of the cells (435, 528, 652, 727, 847 CGG repeats) with the remaining 18% harboring a premutation allele. Sizing of the CGG repeat number by PCR analysis demonstrated the presence of a premutation allele of 90 CGG repeats and an allele with the deletion of the CGG element and the flanking region, the latter of which was not detected by Southern Blot analysis. Sequence analysis of this allele indicated the presence of a deletion extending from nucleotide 13742 to 13915 of the *FMR1 *gene (GenBank accession number L29074). Southern blot analysis of DNA isolated from neural progenitor cells revealed the presence only of hypermethylated full mutation alleles (536 and 591 CGG repeats). No premutation alleles were detected by PCR analysis.

The brain *FMR1 *mRNA level (frontal cortex) relative to the reference gene (glucuronidase) was low (0.08 ± 0.009, relative value). In agreement with the observed lack of FMRP expression, no *FMR1 *message was detected after 40 cycles of PCR in total RNA isolated from the neural progenitor cells.

## Discussion

In the current work, we have successfully isolated and cultured neural progenitor cells from post-mortem brain tissue of an adult male with fragile X syndrome, which, to our knowledge, is the first example of the production of adult, human neural progenitor cells for any neurodevelopmental disorder. This result is of particular importance for the study of fragile X syndrome, since the disease-causing CGG repeat expansions have thus far been refractory to cloning into any animal or human cell model. With these cells, we hope to better understand the mechanistic link between the CGG expansion and the disease phenotype, which is known for the donor of the cells.

We have demonstrated the feasibility of generating neural progenitor cell cultures from post-mortem brains of patients with neurogenetic disease. Moreover, as the cultures were generated from cryopreserved tissue, our data suggest that cells can be harvested and processed from the required tissues in locations that are remote from the stem cell culture laboratory. This last point is of particular importance for the study of neurogenetic diseases, which generally affect only 1:20,000 to 1:200,000 live births; a technique that can be implemented at multiple institutions will be necessary to generate sufficient numbers of specimens for statistical analysis.

A major determinant of the proliferative capacity of neural progenitor cells in culture is donor age, with younger donors (particularly fetuses and infants) having greater proliferative capacity than adults [18]. Although progenitor cells can be obtained from fetal or embryonic sources, there are advantages to obtaining cells from post-mortem adult tissue. In using cells derived from adult tissues, one avoids the serious ethical controversies surrounding the use of fetal samples [25-27]. Moreover, for research aimed at understanding the effects of identified genetic defects on neural development, the phenotypic expression of a particular neurogenetic disease can be ascertained with post-mortem specimens, thus making a correlation possible between *in vitro *and *in vivo *pathophysiology. Due to the broad variability in phenotypic expression in fragile X syndrome (as with many other neurodevelopmental disorders), any such correlations are problematic using tissue obtained at fetopsy.

The method used to isolate the neural progenitor cells in the current study was adapted from the neuro-selective methods developed for culturing CNS stem cells from the brain [28], spinal cord [29], and retina [30] of rodents, as well as the brain of humans [18,20]. Passaged cells expressed a number of immature markers, including the neural stem cell markers CD133 and nestin [20]. Although the currently recognized method for establishing multipotency is clonal derivation followed by differentiation, these cells proliferated poorly when seeded at low density and, therefore, clonal derivation has not been fruitful thus far. Nevertheless, analysis of marker expression provides evidence that these cultures give rise to cells of neuronal lineage (β-III tubulin) and glial lineage (GFAP). Our data are thus most consistent with the interpretation that the immature, highly proliferative neuroepithelial cells in the present study were multipotent neural progenitor cells.

## Conclusions

The successful production of neural cells from an individual with fragile X syndrome opens a new avenue for the scientific study of the molecular basis of this disorder, as well as an approach for studying the efficacy of new therapeutic agents.

## Competing interests

The author(s) declare that they have no competing interests.

## Authors' contributions

PHS conceived of the study, participated in its design and coordination, and drafted the manuscript. FT carried out the molecular genetic studies, participated in the design of the study, and helped to draft the manuscript. CMG performed the neuropathological analyses. HEN carried out the cell culture. BZ performed the immunocytochemical analyses and imaging. RJH performed the clinical evaluation. PJH participated in the design of the study and helped to draft the manuscript. All authors read and approved the final manuscript.

## Pre-publication history

The pre-publication history for this paper can be accessed here:



## References

[B1] Hagerman RJ, Hagerman PJ (2001). Fragile X syndrome: a model of gene-brain-behavior relationships. Mol Genet Metab.

[B2] Hagerman RJ, Hagerman PJ (2002). Fragile X Syndrome: Diagnosis, Treatment, and Research.

[B3] Verkerk AJ, Pieretti M, Sutcliffe JS, Fu YH, Kuhl DP, Pizzuti A, Reiner O, Richards S, Victoria MF, Zhang FP (1991). Identification of a gene (FMR-1) containing a CGG repeat coincident with a breakpoint cluster region exhibiting length variation in fragile X syndrome. Cell.

[B4] Oberlé I, Rousseau F, Heitz D, Kretz C, Devys D, Hanauer A, Boue J, Bertheas MF, Mandel JL (1991). Instability of a 550-base pair DNA segment and abnormal methylation in fragile X syndrome. Science.

[B5] Pieretti M, Zhang FP, Fu YH, Warren ST, Oostra BA, Caskey CT, Nelson DL (1991). Absence of expression of the FMR-1 gene in fragile X syndrome. Cell.

[B6] Antar LN, Bassell GJ (2003). Sunrise at the synapse: the FMRP mRNP shaping the synaptic interface. Neuron.

[B7] Bardoni B, Schenck A, Mandel JL (2001). The Fragile X mental retardation protein. Brain Res Bull.

[B8] The Dutch-Belgian Fragile X Consortium (1994). Fmr1 knockout mice: a model to study fragile X mental retardation. Cell.

[B9] Kooy RF, D'Hooge R, Reyniers E, Bakker CE, Nagels G, De Boulle K, Storm K, Clincke G, De Deyn PP, Oostra BA, Willems PJ (1996). Transgenic mouse model for the fragile X syndrome. Am J Med Genet.

[B10] Dobkin C, Rabe A, Dumas R, El Idrissi A, Haubenstock H, Brown WT (2000). Fmr1 knockout mouse has a distinctive strain-specific learning impairment. Neuroscience.

[B11] Peier AM, McIlwain KL, Kenneson A, Warren ST, Paylor R, Nelson DL (2000). (Over)correction of FMR1 deficiency with YAC transgenics: behavioral physical features. Hum Mol Genet.

[B12] Liu Q, Siomi H, Siomi MC, Fischer U, Zhang Y, Wan L, Dreyfuss G (1996). Molecular characterization of the protein products of the fragile X syndrome gene the survival of motor neurons gene. Cold Spring Harb Symp Quant Biol.

[B13] Morales J, Hiesinger PR, Schroeder AJ, Kume K, Verstreken P, Jackson FR, Nelson DL, Hassan BA (2002). Drosophila fragile X protein, DFXR, regulates neuronal morphology function in the brain. Neuron.

[B14] Svendsen CN, Caldwell MA, Ostenfeld T (1999). Human neural stem cells: isolation, expansion and transplantation. Brain Pathol.

[B15] Temple S, Alvarez-Buylla A (1999). Stem cells in the adult mammalian central nervous system. Curr Opin Neurobiol.

[B16] Gage FH (2000). Mammalian neural stem cells. Science.

[B17] Kukekov VG, Laywell ED, Suslov O, Davies K, Scheffler B, Thomas LB, O'Brien TF, Kusakabe M, Steindler DA (1999). Multipotent stem/progenitor cells with similar properties arise from two neurogenic regions of adult human brain. Exp Neurol.

[B18] Palmer TD, Schwartz PH, Taupin P, Kaspar B, Stein SA, Gage FH (2001). Cell culture Progenitor cells from human brain after death. Nature.

[B19] Pincus DW, Harrison-Restelli C, Barry J, Goodman RR, Fraser RA, Nedergaard M, Goldman SA (1997). In vitro neurogenesis by adult human epileptic temporal neocortex. Clin Neurosurg.

[B20] Schwartz PH, Bryant PJ, Fuja TJ, Su H, O'Dowd DK, Klassen H (2003). Isolation and characterization of neural progenitor cells from post-mortem human cortex. J Neurosci Res.

[B21] Vescovi AL, Parati EA, Gritti A, Poulin P, Ferrario M, Wanke E, Frolichsthal-Schoeller P, Cova L, Arcellana-Panlilio M, Colombo A, Galli R (1999). Isolation and cloning of multipotential stem cells from the embryonic human CNS and establishment of transplantable human neural stem cell lines by epigenetic stimulation. Exp Neurol.

[B22] Vescovi AL, Snyder EY (1999). Establishment and properties of neural stem cell clones: plasticity in vitro and in vivo. Brain Pathol.

[B23] Tamaki S, Eckert K, He D, Sutton R, Doshe M, Jain G, Tushinski R, Reitsma M, Harris B, Tsukamoto A, Gage F, Weissman I, Uchida N (2002). Engraftment of sorted/expanded human central nervous system stem cells from fetal brain. J Neurosci Res.

[B24] Uchida N, Buck DW, He D, Reitsma MJ, Masek M, Phan TV, Tsukamoto AS, Gage FH, Weissman IL (2000). Direct isolation of human central nervous system stem cells. Proc Natl Acad Sci USA.

[B25] Antoniou M (2001). Embryonic stem cell research. The case against. Nat Med.

[B26] Gershon D (2003). Complex political, ethical and legal issues surround research on human embryonic stem cells. Nature.

[B27] McLaren A (2001). Ethical and social considerations of stem cell research. Nature.

[B28] Reynolds BA, Tetzlaff W, Weiss S (1992). A multipotent EGF-responsive striatal embryonic progenitor cell produces neurons and astrocytes. J Neurosci.

[B29] Shihabuddin LS, Ray J, Gage FH (1997). FGF-2 is sufficient to isolate progenitors found in the adult mammalian spinal cord. Exp Neurol.

[B30] Shatos M, Mizumoto K, Mizumoto H, Kurimoto Y, Klassen H, Young M (2001). Multipotent stem cells from the brain and retina of green mice. J Regen Med.

[B31] Tassone F, Hagerman RJ, Garcia-Arocena D, Khandjian EW, Greco CM, Hagerman PJ (2004). Intranuclear inclusions in neural cells with premutation alleles in fragile X associated tremor/ataxia syndrome. J Med Genet.

[B32] Fu YH, Kuhl DP, Pizzuti A, Pieretti M, Sutcliffe JS, Richards S, Verkerk AJ, Holden JJ, Fenwick RG, Warren ST (1991). Variation of the CGG repeat at the fragile X site results in genetic instability: resolution of the Sherman paradox. Cell.

[B33] Tassone F, Hagerman RJ, Taylor AK, Gane LW, Godfrey TE, Hagerman PJ (2000). Elevated levels of FMR1 mRNA in carrier males: a new mechanism of involvement in fragile X syndrome. Am J Hum Genet.

